# Autoimmune Diseases and Risk of Non‐Hodgkin Lymphoma: A Mendelian Randomisation Study

**DOI:** 10.1002/cam4.70327

**Published:** 2024-11-06

**Authors:** Xiaoting Shi, Joshua D. Wallach, Xiaomei Ma, Tormod Rogne

**Affiliations:** ^1^ Department of Environmental Health Sciences Yale School of Public Health New Haven Connecticut USA; ^2^ Yale Center for Perinatal, Pediatric, and Environmental Epidemiology Yale School of Public Health New Haven Connecticut USA; ^3^ Department of Epidemiology, Rollins School of Public Health Emory University Atlanta Georgia USA; ^4^ Department of Chronic Diseases Epidemiology Yale School of Public Health New Haven Connecticut USA; ^5^ Department of Community Medicine and Global Health University of Oslo Oslo Norway

**Keywords:** autoimmune diseases, Mendelian randomisation, non‐Hodgkin lymphoma

## Abstract

**Background:**

Non‐Hodgkin lymphoma (NHL) is one of the most common haematologic malignancies in the world. Despite substantial efforts to identify causes and risk factors for NHL, its aetiology is largely unclear. Autoimmune diseases have long been considered potential risk factors for NHL. We carried out Mendelian randomisation (MR) analyses to examine whether genetically predicted susceptibility to ten autoimmune diseases (Behçet's disease, coeliac disease, dermatitis herpetiformis, lupus, psoriasis, rheumatoid arthritis, sarcoidosis, Sjögren's syndrome, systemic sclerosis, and type 1 diabetes) is associated with risk of NHL.

**Methods:**

Two‐sample MR was performed using publicly available summary statistics from cohorts of European ancestry. For NHL and four NHL subtypes, we used data from UK Biobank, Kaiser Permanente cohorts, and FinnGen studies.

**Results:**

Negative associations between type 1 diabetes and sarcoidosis and the risk of NHL were observed (odds ratio [OR] 0.95, 95% confidence interval [CI]: 0.92–0.98, *p* = 5 × 10^−3^, and OR 0.92, 95% CI: 0.85–0.99, *p* = 2.8 × 10^−2^, respectively). These findings were supported by the sensitivity analyses accounting for potential pleiotropy and weak instrument bias. No significant associations were found between the other eight autoimmune diseases and NHL risk.

**Conclusion:**

These findings suggest that genetically predicted susceptibility to type 1 diabetes, and to some extent sarcoidosis, might reduce the risk of NHL. However, future studies with different datasets, approaches, and populations are warranted to further examine the potential associations between these autoimmune diseases and the risk of NHL.

**Abbreviations**
CI: confidence interval
GWAS: genome‐wide association study
HLA: human leukocyte antigen
IVW: inverse‐variance weighted
MR: Mendelian randomisation
NHL: non‐Hodgkin lymphoma
OR: odds ratio
RAPS: robust adjusted profile scores
SLE: systemic lupus erythematosus
SNP: single‐nucleotide polymorphism
T1D: type 1 diabetes

## 1 Introduction

Non‐Hodgkin lymphoma (NHL), a haematological malignancy that arises from lymphocytes, is one of the most common cancers, with more than 544,000 new cases every year worldwide [1, 2]. Despite substantial efforts to identify risk factors for NHL, the exact aetiology remains elusive [3].

According to a recent umbrella review (i.e., systematic review of meta‐analyses) evaluating the associations between 134 unique environmental risk factors and the risk of NHL, ten autoimmune diseases—Behçet's disease, coeliac disease, dermatitis herpetiformis, psoriasis, rheumatoid arthritis, sarcoidosis, systemic lupus erythematosus (SLE), Sjögren's syndrome, systemic sclerosis, and type 1 diabetes (T1D)—were identified to be statistically significantly associated with an increased risk of NHL [4]. Of these, coeliac disease, rheumatoid arthritis, Sjögren's syndrome, and SLE, were classified as presenting highly suggestive (i.e., *p* < 1 × 10^−6^, at least 1000 NHL cases, and largest study in the review reporting a nominally significant result) or convincing evidence (i.e., *p* < 1 × 10^−6^, at least 1000 NHL cases, largest study in the review reporting a nominally significant result, minimal between‐study heterogeneity, and no evidence of publication bias) evidence for NHL risk [4, 5]. Autoimmune diseases have long been considered potential risk factors for NHL [6, 7]. Proposed mechanisms for the associations between autoimmune diseases and NHL include chronic inflammation, antigen stimulation, and overlapping genetic susceptibility [8, 9, 10]. However, the associations identified by the umbrella review were from systematic reviews and meta‐analyses with various study design and reporting limitations [4, 5]. Furthermore, the systematic reviews and meta‐analyses included only case–control and cohort studies, which are susceptible to multiple biases that limit their ability to evaluate causal relationships. For instance, confounding factors (e.g., socioeconomic status, family history of lymphoma, and infectious diseases) and reverse causation were often not considered by the previous studies [11, 12]. For some autoimmune diseases, such as sarcoidosis, it is often unclear if the autoimmune disease precedes or develops after NHL [13, 14, 15, 16].

One way of addressing the issue of residual confounding and reverse causation is through instrumental variable analyses with genetic instruments, often called Mendelian randomisation (MR) analyses [17]. Because genetic variants are randomly assigned at conception and are not affected by external factors such as chronic diseases and lifestyle factors, MR analyses mimic randomised experiments and are less susceptible to confounding and reverse causation compared with conventional observational studies [18].

Therefore, the aim of this study was to use the MR design to evaluate the associations between genetically predicted susceptibility to ten autoimmune diseases and the risk of NHL and NHL subtypes (follicular lymphoma, mature T/natural killer‐cell lymphomas, non‐follicular lymphoma, and other and unspecified types of NHL) [4]. Because nearly all observational studies on the associations between autoimmune diseases and NHL suggested a positive association [4, 9, 11, 12], our hypothesis was that the genetically predicted susceptibility to each autoimmune disease was associated with an increased risk of NHL and at least one of the NHL subtypes.

## 2 Methods

This study is reported following the Strengthening the Reporting of Observational Studies in Epidemiology Using Mendelian Randomisation guidelines (STROBE‐MR, Supporting Information) [19]. Although there is no pre‐registered protocol for this study, the analyses were designed prior to the conduct of the study. Data were retrieved between June 2022 and June 2023, and analyses were conducted between June 2022 and October 2023. The manuscript was posted on *medRxiv* (doi: https://doi.org/10.1101/2024.01.20.24301459). Only summary‐level data from published studies with relevant ethical approvals were used in this study, so approval from institutional review board was not necessary.

### 2.1 Study Design

**FIGURE 1:**
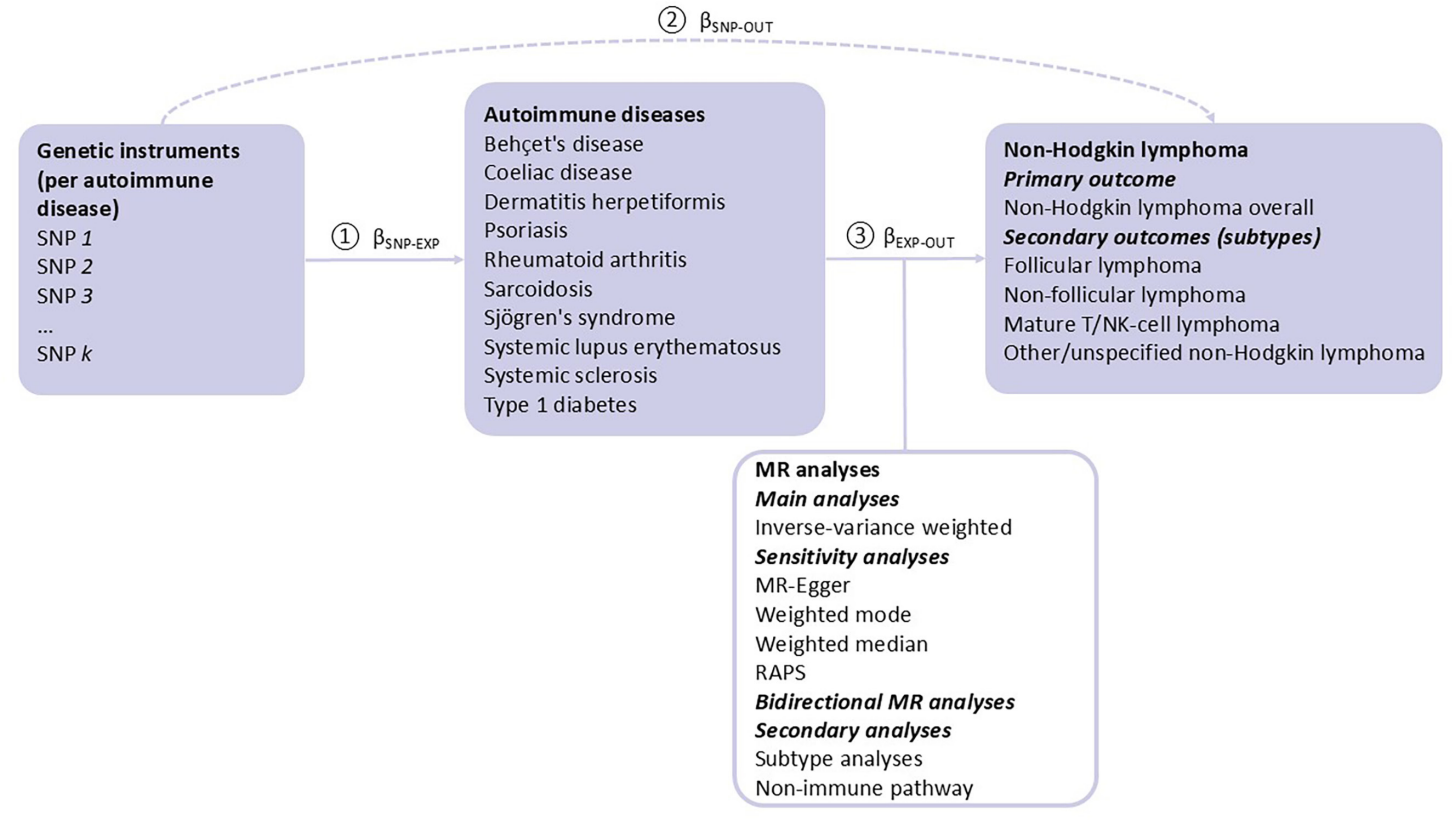
A schematic overview of the study design. EXP, exposure; MR, Mendelian randomisation; OUT, outcome; RAPS, robust adjusted profile scores; SNP, single‐nucleotide polymorphism. Wald ratio: β_EXP‐OUT_ $\;=\frac{\text{βSNP}-\text{OUT}}{\text{βSNP}-\mathrm{EXP}}$; The analyses were carried out in three steps: Step 1, genetic instruments for each autoimmune disease were selected from relevant GWASs (i.e., *p* < 5 × 10^−8^ and an *R* ^2^ < 0.001), with *β*_SNP‐EXP_ (i.e., association between genetic instrument and the exposure) extracted. Step 2, for these genetic instruments, the genetic instrument‐outcome associations *β*_SNP‐OUT_ were extracted from the outcome GWAS. Step 3, for each autoimmune disease, the Wald ratio was estimated for *k* number of SNPs by the formula *β*_EXP‐OUT_ = *β*_SNP‐OUT_/*β*_SNP‐EXP_, and next summarised using the listed MR analyses. In the secondary analyses, only inverse‐variance weighted method was applied.

Figure 1 shows a schematic summary of this two‐sample MR study, a design that has been widely applied in the setting of dichotomous exposures and risk of a dichotomour outcome (as in our case) [17]. Overall, we extracted summary level data from genome‐wide association studies (GWASs) to find genetic instruments for our exposures of interest (i.e., exposure GWASs) and then investigated the associations of these genetic instruments with the outcomes of interest (i.e., outcome GWASs) [17]. For the genetic instruments to be valid, three core assumptions must be met: [20] (1) the instruments are associated with the exposure of interest (the relevance assumption), (2) the instruments are not associated with any confounders of the exposure‐outcome relationship (the independence assumption), and (3) the instruments are associated with the outcome only through the exposure (the exclusion restriction assumption).

The analyses were carried out in three steps: first, genetic instruments for each exposure of interest (i.e., autoimmune disease) were selected from relevant exposure GWASs. Second, for these genetic instruments, the genetic instrument‐outcome associations were extracted from the relevant outcome GWASs. For each autoimmune disease, the exposure‐outcome association (i.e., the Wald ratio) was estimated by dividing the genetic instrument‐outcome association by the genetic instrument‐exposure association. Finally, the Wald ratios were summarised using different techniques (See Section 2.4). Due to data availability and to minimise confounding due to population stratification, we only considered subjects of European ancestry [17].

### 2.2 Instrumental Variable Selection for Autoimmune Diseases

To fulfil the relevance assumption, we used single‐nucleotide polymorphisms (SNPs) as genetic instruments and selected those from GWASs that were (1) strongly associated with the specific autoimmune disease at genome‐wide significance (i.e., *p* < 5 × 10^−8^) and (2) independent of one‐another (i.e., an *R* ^2^ < 1 × 10^−3^). We used a less stringent criterion for significance level, *p* < 5 × 10^−6^ for dermatitis herpetiformis and Sjögren's syndrome to ensure that we had at least five SNPs as genetic instruments for each autoimmune disease.

We evaluated the ten autoimmune diseases that were at least nominally significantly associated (i.e., *p* < 5 × 10^−2^) with risk of NHL according to a recent umbrella review: [4] Behçet disease, coeliac disease, dermatitis herpetiformis, psoriasis, rheumatoid arthritis, sarcoidosis, Sjögren's syndrome, SLE, systemic sclerosis, and T1D. These autoimmune diseases were all associated with an increased risk of NHL. Of these, coeliac disease, rheumatoid arthritis, Sjögren's syndrome, and SLE, were classified as having highly suggestive (i.e., *p* < 1 × 10^−6^, at least 1000 NHL cases, and largest study in the review reporting a nominally significant result) or convincing evidence (i.e., *p* < 1 × 10^−6^, at least 1000 NHL cases, largest study in the review reporting a nominally significant result, minimal between‐study heterogeneity, and no evidence of publication bias) in the umbrella review [4]. For each autoimmune disease, we selected the GWAS that had the largest sample size. In situations where no distinct GWAS publication for an autoimmune disease was available, we used summary statistics from genome‐wide association analyses from FinnGen Release 8 (https://r8.finngen.fi/, Supporting Information) [21]. Study characteristics of all summary level GWAS datasets for the autoimmune diseases used in main analyses are shown in Table 1.

**TABLE 1:** GWAS datasets used for the autoimmune diseases in the in main analyses.

Trait	Study	Countries	Cohorts	Number of cases	Number of controls	Number of SNPs	*R* ^2^ (% of variance explained)	Median *F* statistics (range)
Behçet disease	Fernández et al. 2021 [43]	West Europe, Italy, Spain	Meta‐analysis of three independent cohorts	437	3325	7	0.3	1 (0.1–7.9)
Coeliac disease	Dubois et al. 2010 [44]	The UK, the USA, Finland, Italy, Netherlands, Poland, Hungary, Spain	Meta‐analysis of seven independent cohorts	4918	5684	12	1.2	53 (30–96)
Dermatitis herpetiformis	Kurki et al. 2023 [21]	Finland	FinnGen	435	341,188	8*	1.3	22 (20–35)
Psoriasis	Tsoi et al. 2017 [45]	North America, Sweden	Meta‐analysis of eight independent cohorts	11,988	275,335	32	1.8	59 (30–310)
Rheumatoid arthritis	Ishigaki et al. 2022 [46]	The US, the UK, Canada, Spain, Sweden, the Netherlands, France	Meta‐analysis of 25 independent cohorts	22,350	74,823	59	2.4	42 (30–762)
Sarcoidosis	Kurki et al. 2023 [21]	Finland	FinnGen Study	3597	337,121	9	0.7	34 (32–116)
Sjögren's syndrome	Kurki et al. 2023 [21]	Finland	FinnGen Study	2735	332,115	11*	0.8	23 (21–73)
Systemic lupus erythematosus	Wang et al. 2021 [47]	Spain, northern and western Europe, Italy	Meta‐analysis of three independent cohorts	4576	8039	24	2.5	44 (30–268)
Systemic sclerosis	López‐Isac et al. 2019 [48]	Spain, Germany, the Netherlands, USA, France, Spain, Italy, UK, Sweden, Norway, Australia/UK	Meta‐analysis of 14 independent cohorts	9095	17,584	21	1.3	41 (30–100)
Type I diabetes	Chiou et al. 2021 [49]	The USA, the UK, Ireland, Finland	Meta‐analysis of nine independent cohorts including FinnGen Study	18,942	501,638	72	3.1	45 (30–1077)

Abbreviations: GWAS, genome‐wide association study; ICD‐10, International Classification of Diseases, Tenth Revision; NA, not available; the UK, the United Kingdoms; the USA, the United States.

* *p* < 5 × 10^−6^ was used as criteria to select genetic instruments.

Because human leukocyte antigen (HLA) region is highly predictive of both autoimmune diseases and NHL, we excluded SNPs from this locus from all analyses to prevent potential bias due to genetic pleiotropy [22, 23]. We defined the HLA region as location from 28,477,797 to 33,448,354 on chromosome 6 for Genome Reference Consortium Human Build 37 [24].

### 2.3 Outcomes

Our primary outcome was risk of NHL. We selected the largest GWAS on NHL, which evaluated subjects from UK Biobank and Kaiser Permanente (Table 2, Supporting Information) [25]. In secondary analyses for the genetically‐predicted autoimmune diseases found to be nominally significantly associated with NHL (see Section 2.4), we further evaluated the associations between these autoimmune diseases and NHL subtypes. We identified the NHL subtypes from FinnGen Release 8 and we selected four major NHL subtypes, consistent with the FinnGen classification system: follicular lymphoma, mature T/natural killer‐cell lymphomas, non‐follicular lymphoma, and other and unspecified types of NHL (Table 2) [21]. Of note, as the data on the NHL subtypes were extracted from a different dataset from the NHL GWAS, the secondary analyses also served as replication analyses using an independent dataset [21].

**TABLE 2:** GWAS datasets used for the non‐Hodgkin lymphoma in the in main analyses.

Trait	GWAS data source	Countries	Cohorts	Case definition	Control definition	Number of cases	Number of controls
Non‐Hodgkin lymphoma	Rashkin et al. 2020 [25]	The UK, the USA	UK Biobank, Kaiser Permanente cohorts	ICD‐O‐3 codes and SEER cite recode paradigm	Individuals with no record of any cancer in the relevant registries	2400	410,350
Follicular lymphoma	Kurki et al. 2023 [21]	Finland	FinnGen Study	ICD‐10: C82	Population controls	955	271,463
Mature T/NK‐cell lymphomas	Kurki et al. 2023 [21]	Finland	FinnGen Study	ICD‐10: C84	Population controls	296	271,463
Non‐follicular lymphoma	Kurki et al. 2023 [21]	Finland	FinnGen Study	ICD‐10: C83	Population controls	2340	271,463
Other and unspecified types of non‐Hodgkin lymphoma	Kurki et al. 2023 [21]	Finland	FinnGen Study	ICD‐10: C85	Population controls	982	271,463

Abbreviations: GWAS, genome‐wide association study; ICD‐10, International Classification of Diseases, Tenth Revision; NA, not available; the USA, the United States; UK, United Kingdoms.

### 2.4 Statistical Analyses

#### 2.4.1 Main Analyses and Sensitivity Analyses

For each autoimmune disease, we calculated the SNP‐specific Wald ratio, defined as β_EXP‐OUT_ = *β*_SNP‐OUT_/*β*_SNP‐EXP_ (Figure 1). We used inverse‐variance weighted (IVW) analysis as our main analyses to sum the Wald ratios, which assign weights to each SNP in inverse proportion to the variance of the *β*_SNP‐OUT_, assuming all instruments to be valid [26, 27]. However, the IVW analysis may be biased if any of the included instruments are invalid (e.g., if the genetic instruments affect multiple traits, which is known as horizontal pleiotropy) [28]. Therefore, we carried out three sensitivity analyses that provide unbiased estimates even in the presence of some invalid instruments: [17] MR‐Egger regression [27], weighted mode estimator analysis [29], and weighted median estimator analysis (Supporting Information) [30]. Furthermore, to address potential weak instrument bias, which may be introduced when the genetic variants explain a very small proportion of the variation in the exposure [20], we included robust adjusted profile scores (RAPS) as an additional sensitivity analysis (Supporting Information) [17, 31]. Lastly, for each autoimmune disease, leave‐one‐out analyses were performed to test whether single SNPs had an outsized effect on the overall association between the autoimmune disease and risk of NHL [27].

We evaluated the strength of the instruments using *R* ^2^ (i.e., proportion of variance of the exposure explained by the genetic instrument) and *F* statistics. In particular, we used the *get_r_from_bsen* function in the *TwoSampleMR* package in R (version 4.3.0), and summed the absolute values across the independent SNPs to estimate the composite *R* ^2^ for each autoimmune disease. *F* statistics were calculated using the formula *F* statistic $={\left(\frac{{\hat{\beta }}_{X}}{\mathrm{se}\left({\hat{\beta }}_{X}\right)}\right)}^{2}$ [32], where ${\hat{\beta }}_{X}$ refers to the genetic association between the instrument *X* with the exposure, and $\mathrm{se}\left({\hat{\beta }}_{X}\right)$ refers to the standard error of ${\hat{\beta }}_{X}$.

#### 2.4.2 Bidirectional Analyses

To help establish the direction of effects between two traits [17], we carried out bidirectional analyses of genetically predicted NHL and risk of each autoimmune disease [17]. Evidence of an effect in both directions could suggest that an effect acts in both directions between two traits so that changing one will change the other (i.e., the true bidirectional relationship). Otherwise it may suggest that a biasing pathway may be present [17]. Evidence of an effect in one direction but not the other supports that a biasing pathway is less likely to be present.

#### 2.4.3 Secondary Analyses

To further validate the principal findings from the main analyses, we carried out two sets of secondary analyses for the autoimmune diseases that were nominally significantly associated with risk of NHL (i.e., T1D and sarcoidosis, see Section 3): (1) evaluating the risk of four NHL subtypes using a dataset independent from the main analyses and (2) restricting the genetic instruments to those that are less likely to affect immune function. The analyses of the NHL subtypes were carried out to identify which of the NHL subtypes were driving the observed association with NHL, and also to serve as a replication using a separate outcome study population not overlapping with the NHL study population of the other analyses. The analysis involving selected pathways were carried out to address horizontal pleiotropy. In particular, given the shared role of immune function in the pathway to developing both autoimmune diseases and NHL, we sought to identify biological pathways for the autoimmune diseases that were to a lesser degree linked to immune function (Table S1). For T1D, we restricted this secondary analysis to SNPs linked to insulin production, whereas for sarcoidosis we selected SNPs that were not directly linked to immune function. The KEGG and GeneCards databases were used to identify the function of all T1D and sarcoidosis SNPs [33, 34]. For all secondary analyses, only IVW analyses were carried out.

#### 2.4.4 Software

We used *TwoSampleMR* package in R (version 4.3.0) to run the two‐sample MR analyses, and *forplo* to generate the forest plots [35]. A *p* < 5 × 10^−2^ was considered nominally significant. To correct for multiple testing of the ten autoimmune diseases in the main analyses, the level for statistical significance was set at *p* < 5 × 10^−2^/10 = 5 × 10^−3^. Statistical significance was not considered in the sensitivity and secondary analyses, but the results were qualitatively compared with the corresponding main analysis. To make the results more interpretable, all causal estimates were multiplied by 0.693 (= $\;\log_{e}^{2}$) and next exponentiated to represent the odds ratios (ORs) for NHL per doubling in the prevalence of the autoimmune disease under study [32].

## 3 Results

The number of cases and controls identified in the relevant GWASs were 437 and 3325 for Behçet's disease, 4918 and 5684 for coeliac disease, 435 and 341,188 for dermatitis herpetiformis, 4576 and 8039 for lupus, 11,988 and 275,335 for psoriasis, 22,350 and 74,823 for rheumatoid arthritis, 3597 and 337,121 for sarcoidosis, 2735 and 332,115 for Sjögren's syndrome, 9095 and 17,584 for systemic sclerosis, 18,942 and 501,638 for type 1 diabetes, and 2400 and 410,350 for NHL (Tables 1 and 2) [25]. The variance in the exposure explained by the genetic instruments ranged from 0.3% for Behçet disease to 3.1% for T1D (Table 1).

### 3.1 Primary Analyses

**FIGURE 2:**
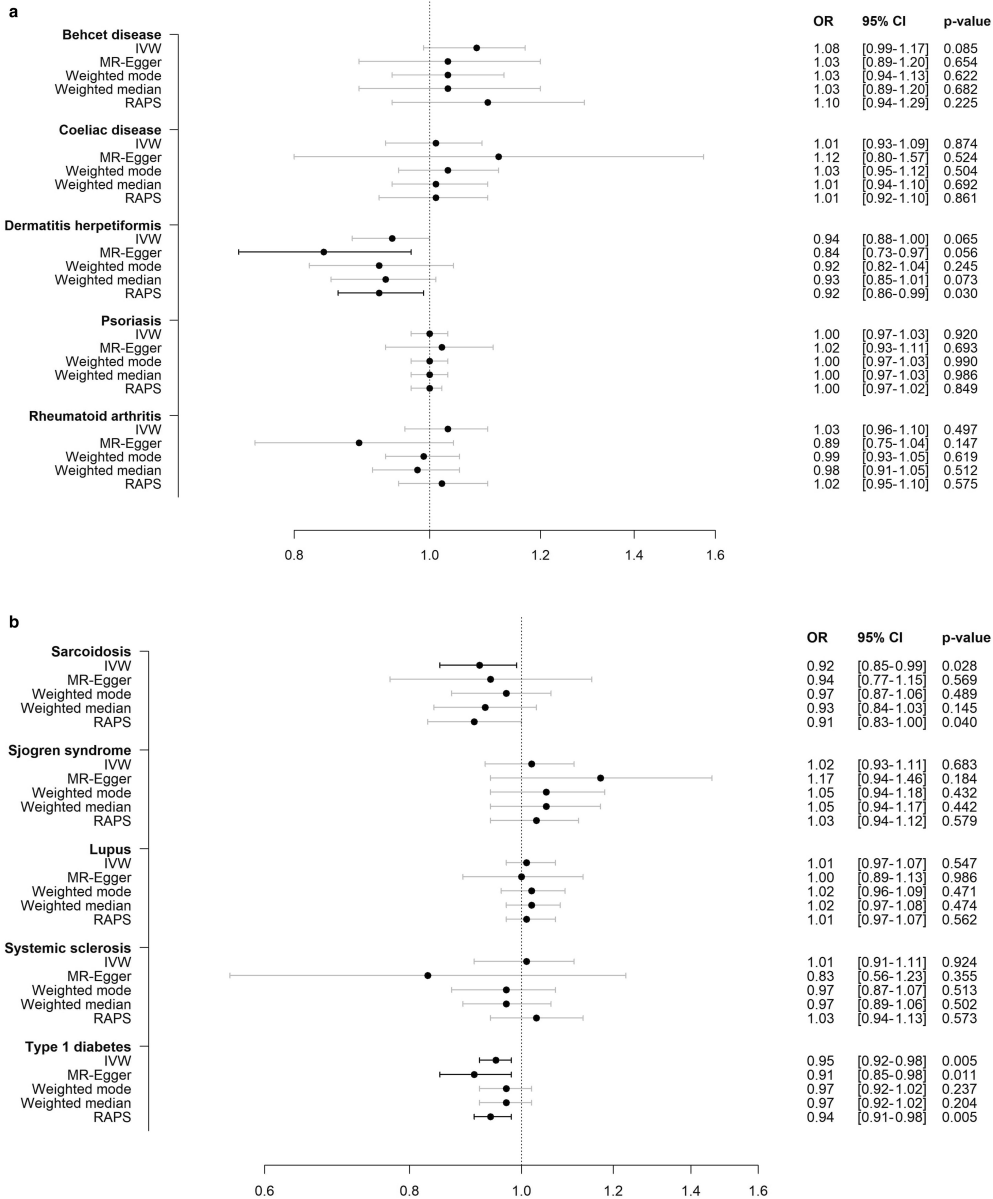
(a and b) Genetically predicted susceptibility to ten autoimmune diseases and risk of non‐Hodgkin lymphoma. CI, confidence interval; IVW, inverse‐variance weighted; MR, Mendelian randomisation; OR, odds ratio; RAPS, robust adjusted profile scores. All estimates were multiplied by 0.693 and next exponentiated to represent the ORs for NHL per doubling in the prevalence of the autoimmune disease under study.

A doubling in the genetically‐predicted prevalence of T1D was associated with an OR for NHL of 0.95 (95% confidence interval [CI]: 0.92–0.98, *p* = 5 × 10^−3^), whereas a doubling in the genetically‐predicted prevalence of sarcoidosis was associated with an OR for NHL of 0.92 (95% CI: 0.85–0.99, *p* = 2.8 × 10^−2^) (Figure 2). We did not observe significant associations between the other eight autoimmune diseases and risk of NHL (Figure 2).

### 3.2 Sensitivity Analyses

MR‐Egger, weighted mode and weighted median yielded ORs comparable to those from the main analyses, and with overlapping CIs, indicating little presence of pleiotropy (Figure 2). Furthermore, the RAPS sensitivity analyses did not suggest bias due to weak instruments. For the bidirectional analyses we did not observe significant associations between NHL and the risk of any of the autoimmune diseases (Table S2). The results from the leave‐one‐out analyses indicated that the associations between the autoimmune diseases and risk of NHL were not driven by any single SNPs independently of the other SNPs (Figures S1–S10).

### 3.3 Secondary Analyses

In the secondary analyses, we evaluated the autoimmune diseases that were at least nominally significantly associated with risk of NHL, i.e., T1D and sarcoidosis. In the analyses restricted to non‐immune pathways, we observed ORs of 0.96 (95% CI: 0.87–1.07) for T1D and 0.96 (95% CI: 0.88–1.06) for sarcoidosis, respectively, supporting the main analyses.

**FIGURE 3:**
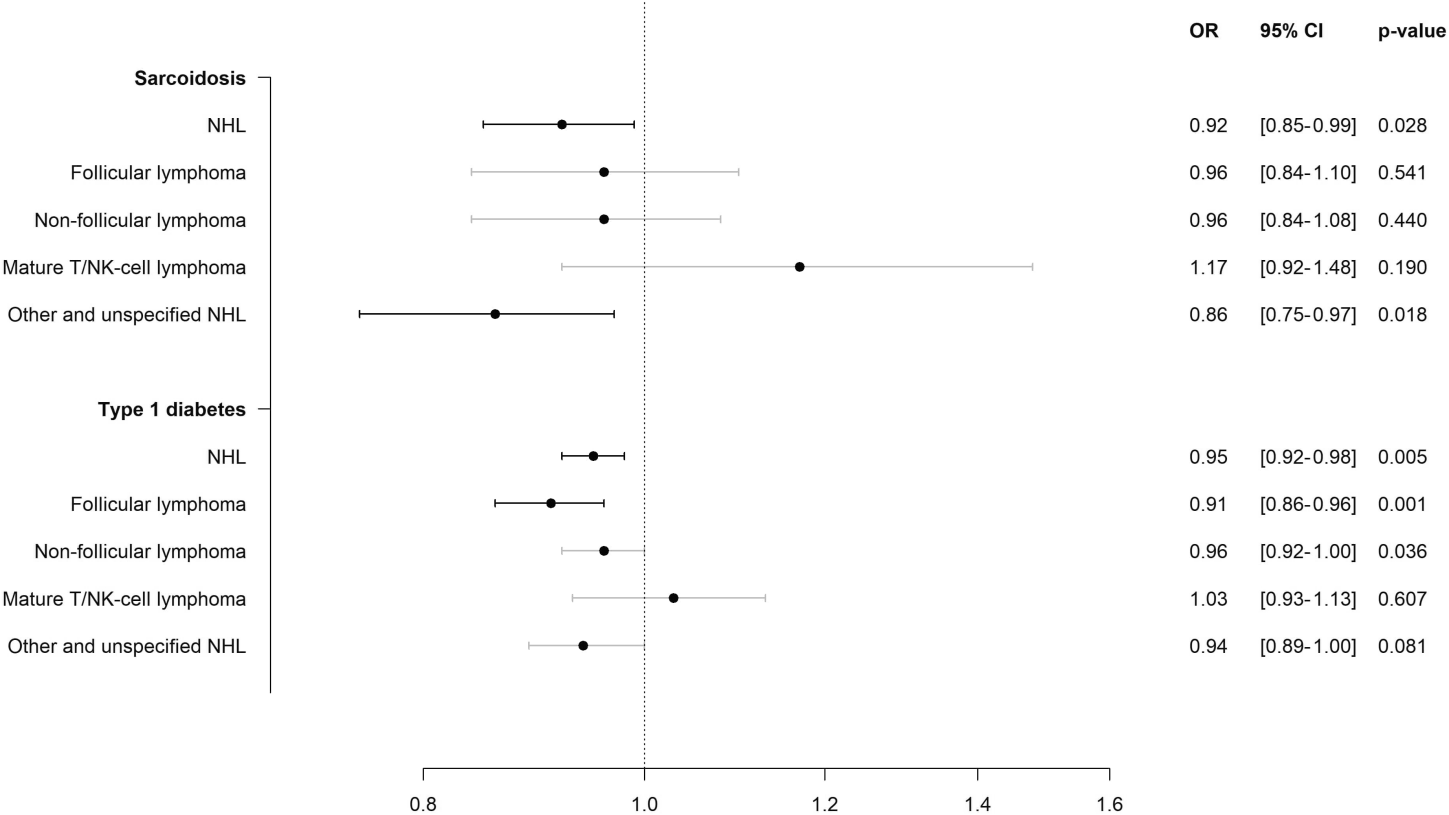
Inverse‐variance weighted analyses of genetically predicted susceptibility to sarcoidosis and type 1 diabetes, and risk of non‐Hodgkin lymphoma subtypes. CI, confidence interval; NHL, non‐Hodgkin lymphoma; OR, odds ratio. The analyses were based on inverse‐variance weighted approach. All estimates were multiplied by 0.693 and next exponentiated to represent the odds ratios (ORs) for NHL per doubling in the prevalence of the autoimmune disease under study. Different datasets were used for NHL and NHL subtypes.

Figure 3 shows the IVW analyses of genetically predicted susceptibility to T1D and sarcoidosis, and the risk of NHL subtypes. For T1D, the association with composite NHL appeared to be driven by follicular lymphoma, with an OR of 0.91 (95% CI: 0.86–0.96, *p* = 1 × 10^−3^). Sarcoidosis was most strongly associated with other and unspecified types of NHL, with an OR of 0.86 (95% CI: 0.75–0.97, *p* = 1.8 × 10^−2^).

## 4 Discussion

### 4.1 Principal Findings

In this MR study of ten autoimmune diseases previously linked to an increased risk of NHL, we found that genetically predicted susceptibility to T1D, and to some extent, genetically predicted susceptibility to sarcoidosis, were associated with a reduced risk of NHL. Although these findings were consistent across a wide range of sensitivity analyses, no clear associations were observed between the other eight autoimmune diseases and risk of NHL. Using an approach that attempts to address potential residual confounding and reverse causation, our findings contradict those reported in previous traditional observational studies. This highlights the need for future studies with different datasets, approaches, and populations to further examine the potential associations between these autoimmune diseases and the risk of NHL.

### 4.2 Context of Primary Findings

Autoimmune diseases have long been considered potential risk factors for NHL, especially rheumatoid arthritis, SLE, and Sjögren's syndrome [6, 7, 12, 36]. A recent large‐scale prospective cohort study found that among all cancers, lymphoma demonstrated the most extensive associations with different immune‐mediated diseases [9]. According to a previous umbrella review evaluating the associations between any environmental risk factors and the risk of NHL reported in published meta‐analyses, there were consistent statistically significant associations between autoimmune diseases and NHL risk [4]. Although the exact mechanisms for positive associations between autoimmune diseases and NHL remain unclear, there are several mechanisms proposed, including chronic inflammation, antigen stimulation, overlapping genetic susceptibility, and dysfunction of certain protein families [8, 9, 10]. Furthermore, the observed associations between autoimmune diseases and NHL may be attributable to the immunosuppressants that are used as treatments as well [36].

In our study, two autoimmune diseases ‐T1D and sarcoidosis—were found to be associated with NHL. However, contrary to our pre‐specified hypotheses, which were based on the summary relative risks from an umbrella review (relative risk 1.55 [95% CI: 1.15–2.08] for associations between T1D and NHL, and relative risk 1.43 [95% CI: 1.03–1.99] for sarcoidosis and NHL) [4], we found a statistically significant negative association between genetically predicted susceptibility to T1D and NHL. Although we also observed a negative association between genetically predicted susceptibility to sarcoidosis and NHL, the association was no longer significant after accounting for multiple testing. Although no previous epidemiologic studies have reported statistically significant negative associations for either T1D or sarcoidosis with NHL [8, 9, 12, 16, 37], it has been suggested that the pathways involved in autoimmune disease and cancer development may work in opposite directions [38]. Moreover, it remains unclear if sarcoidosis precedes or follows NHL, as several observational studies reported occurrence of sarcoidosis among NHL patients after treatment of NHL or concomitant occurrence of two diseases [13, 14, 15, 16].

There are several potential explanations for the discrepancy between our findings and those of the previous umbrella review. First, meta‐analyses of observational studies and MR analyses have different bias structures. Although traditional observational studies often suffer from confounding, misclassification, and selection bias, and meta‐analyses are often susceptible to publication bias (i.e., the lack of publishing certain findings, often null findings), horizontal pleiotropy is of particular concern for MR studies. Second, many of the traditional observational studies and meta‐analyses have relatively small sample sizes. In particular, the previous meta‐analyses for T1D only included three individual observational studies with 1155 NHL cases and the meta‐analysis for sarcoidosis only included seven studies with 150 sarcoidosis cases [4, 39]. Furthermore, according to a formal critical appraisal tool for systematic reviews and meta‐analyses (i.e., AMSTAR 2), both meta‐analyses were found to have at least one critical weakness [4, 5]. Third, given that genetic variants are fixed at conception, the exposure measured in MR analyses are typically interpreted as the lifelong exposure. In observational studies on the association between autoimmune diseases and NHL, the exposure is usually measured and interpreted in a defined time period from disease onset of autoimmune diseases. Finally, we conducted our study in population of European ancestry, whereas some meta‐analyses investigated the associations in multiple populations other than European ancestry [39, 40]. However, the findings from populations with different ancestries did not differ drastically in the umbrella review [39, 40].

### 4.3 Strengths and Limitations of This Study

The MR design is a major strength of our study, as it reduces the influence of residual confounding and reverse causation. In particular, confounding due to infectious diseases (an important risk factor for NHL and for autoimmune diseases) is minimised in this study. Moreover, to our knowledge, this is the first MR study that investigates the association between multiple autoimmune diseases and NHL.

Our study had several limitations. First, this study is limited to populations of European ancestry, which have the highest incidence of NHL, and it is unclear whether the findings can be generalised beyond this population. However, only GWAS data for populations of European ancestry are publicly available for all the autoimmune diseases, NHL, and NHL subtypes that we investigated [3]. Furthermore, using the same population also ensures that the exposure dataset and outcome dataset are as similar as they could be. Second, NHL is a heterogenous group of haematologic disorders, with more than 20 subtypes [41]. Although we used the most robust dataset for NHL subtypes (i.e., FinnGen), different categorisation of NHL subtypes is possible and may yield different results. Third, one autoimmune disease, Behçet disease, had suboptimal *F* statistics for its genetic instruments, which may cause weak instrument bias. However, when we conducted RAPS analysis to address potential bias due to weak instruments, the observed findings were similar to the main analyses [17, 31]. Lastly, given the shared genetic basis of autoimmune diseases and NHL [10, 42], we were particularly cautious about potential bias due to horizontal pleiotropy by immune function. To minimize the impact of this bias, we excluded SNPs in the HLA region and carried out various sensitivity analyses. We also carried out secondary analyses for T1D and sarcoidosis restricted to the insulin and non‐immune pathways, respectively. Although our sensitivity analyses did not suggest important bias due to genetic pleiotropy, it is still possible that potential pleiotropy may partially affect our study findings.

Although our study was designed to address confounding and reverse causation, we emphasise that this MR study alone does not provide conclusive causal estimates. Therefore, the reported associations need further investigation with evidence triangulation using different datasets, populations, and approaches.

## 5 Conclusions

This MR analysis found that genetically predicted susceptibility to T1D, and to some extent genetically predicted susceptibility to sarcoidosis, was associated with a lower risk of NHL. Future research using different datasets, approaches, and populations is necessary to develop a more comprehensive understanding of the associations between T1D and NHL and sarcoidosis and NHL.

## Author Contributions

**Xiaoting Shi:** conceptualization (equal), data curation (equal), formal analysis (lead), methodology (equal), visualization (equal), writing – original draft (lead), writing – review and editing (equal). **Joshua D. Wallach:** conceptualization (equal), funding acquisition (equal), investigation (equal), methodology (equal), project administration (equal), resources (equal), supervision (equal), validation (equal), visualization (equal), writing – review and editing (equal). **Xiaomei Ma:** methodology (equal), resources (equal), writing – review and editing (equal). **Tormod Rogne:** conceptualization (equal), data curation (equal), funding acquisition (lead), investigation (equal), methodology (equal), project administration (lead), resources (equal), supervision (lead), validation (equal), visualization (equal), writing – review and editing (equal).

## Conflicts of Interest

All authors have completed the ICMJE uniform disclosure form at www.icmje.org/coi_disclosure.pdf and declare: In the past 36 months, Dr. Wallach reported receiving grant support from the FDA, Arnold Ventures, Johnson & Johnson through Yale University, and the National Institute on Alcohol Abuse and Alcoholism of the National Institutes of Health (NIH) under award 1K01AA028258; serving as a consultant for Hagens Berman Sobol Shapiro LLP and Dugan Law Firm APLC; and serving as a *medRxiv* affiliate. Dr. Ma received research funding from the NIH and the Frederick A. DeLuca Foundation and served as a consultant for Bristol Myers Squibb.

## Supporting information


Data S1.

**Data S2.** STROBE‐MR checklist of recommended items to address in reports of Mendelian randomisation studies1 2.

Data S3.

## Data Availability

All the SNPs used in the study are provided in Supporting Information. This work has been conducted using different cohorts from including UK Biobank, Kaiser Permanente cohorts, and FinnGen studies. The bona fide researchers can use the dataset by registering for and applying for the above‐mentioned studies. All source code is publicly available on Open Science Framework (https://osf.io/kz35q/) and in Table S3. Further information is available from the corresponding author upon request.
